# Population knowledge on chronic kidney disease, its risk factors and means of prevention: a population-based study in Fortaleza, Ceará, Brazil

**DOI:** 10.1590/2175-8239-JBN-2022-0017en

**Published:** 2022-09-30

**Authors:** Ana Carolina Rattacaso Marino de Mattos Albuquerque, Gustavo Neves Pinto, Gabriel Araújo Pereira, Luísa Falcão Silva, Thaís Azevedo Souza Fontenele, Juliana Gomes Ramalho de Oliveira, Geraldo Bezerra da Silva

**Affiliations:** 1 Universidade de Fortaleza, Programa de Pós-Graduação em Saúde Coletiva, Fortaleza, CE, Brasil.; 2 Universidade de Fortaleza, Curso de Medicina, Centro de Ciências da Saúde, Fortaleza, CE, Brasil.; 3 Universidade Federal do Ceará, Faculdade de Medicina, Departamento de Medicina Clínica, Fortaleza, CE, Brasil.

**Keywords:** Renal Insufficiency, Chronic, Disease Prevention, Knowledge, Population Characteristics, Insuficiência Renal Crônica, Prevenção de Doenças, Conhecimento, Características da População

## Abstract

**Introduction:**

Chronic kidney disease (CKD) has been increasing significantly. There is evidence that a large part of the population does not have enough knowledge on the subject.

**Objective:**

To investigate the level of knowledge about CKD in the general population, its risk factors and means of prevention.

**Methods:**

We ran a cross-sectional study in the population of Fortaleza, Ceará – Brazil, between 2017 and 2020, with the application of a questionnaire on CKD, risk factors and prevention.

**Results:**

we interviewed 735 volunteers, with a mean age of 38 years, of which 55% were female. Only 17.2% correctly responded to the concept of CKD, and 5.8% knew the concept of creatinine. Low water intake was the most cited risk factor by respondents (79.3%). The main risk factors and direct causes of CKD (diabetes and hypertension) were mentioned less frequently (13.2% and 15.1%, respectively). Men were more correct regarding risk factors and ways to prevent CKD. Older respondents answered more correctly the questions about the definition of CKD (n = 22; 28.6%) and creatinine (n = 7; 9.0%). With regards to education there was a statistically significant correlation in all the questions (p < 0.05).

**Conclusion:**

There is little knowledge about CKD in the general population. Higher level of education is associated with better knowledge. More health education actions are needed so that the population becomes better acquainted with CKD and, consequently, can adopt more adequate prevention and control measures.

## Resumo

**Introdução:**

A doença renal crônica (DRC) vem aumentando de forma significativa. Há evidências de que grande parte da população não tem conhecimento suficiente sobre o assunto. Objetivo: Investigar na população geral o nível de conhecimento sobre a DRC, seus fatores de risco e meios de prevenção.

**Métodos:**

Foi realizado estudo transversal na população de Fortaleza, Ceará, Brasil, entre 2017 e 2020, com aplicação de um questionário sobre DRC, fatores de risco e prevenção.

**Resultados:**

Foram entrevistados 735 voluntários, com média de idade de 38 anos, dos quais 55% eram do sexo feminino. Apenas 17,2% responderam corretamente ao conceito de DRC, e 5,8% sabiam o conceito de creatinina. A baixa ingestão hídrica foi o fator de risco mais citado pelos entrevistados (79,3%). Os principais fatores de risco e as causas diretas de DRC (diabetes e hipertensão) foram mencionados com menor frequência (13,2% e 15,1%, respectivamente). Os homens tiveram maior acerto com relação aos fatores de risco e às formas de prevenção da DRC. Os entrevistados com idade mais avançada responderam mais corretamente às perguntas sobre a definição de DRC (n = 22; 28,6%) e creatinina (n = 7; 9,0%). No que diz respeito à escolaridade, em todas as perguntas houve correlação estatisticamente significativa (p < 0,05).

**Conclusão:**

Há um baixo nível de conhecimento sobre a DRC na população geral. Maior nível de escolaridade associa-se a um melhor conhecimento. São necessárias mais ações de educação em saúde para que a população conheça melhor a DRC e consequentemente possa adotar medidas de prevenção e controle mais adequadas.

## Introduction

Chronic kidney disease (CKD) consists of a gradual and irreversible decline in kidney function for 3 months or more, involving the glomerulus, tubules and their endocrine action/ being clinically identified by a reduction in the glomerular filtration rate of less than 60 mL/min/1.73 m^2^; and/or increased urinary albumin excretion^1,2^.

In its early stages CKD is asymptomatic, and this makes its early detection difficult. Thus, it progresses slowly, and the identification of the disease usually only occurs in stages with significant loss of renal function. Incorrect diagnosis delays referral to nephrologists, which results in lost time and opportunity to implement strategies that delay disease progression^1,3,4^. One of the first studies to present the effects of late referral to the nephrologist was by Ratcliffe et al.^5^, in England, which even showed higher mortality among patients who started hemodialysis later. In Brazil, one of the first evidence of late referral to a nephrologist was published by Sesso et al.^6^, who showed that more than 70% of patients who had started dialysis at a referral center in São Paulo had not consulted a nephrologist before starting the dialysis treatment, and 41% of the patients were diagnosed with CKD less than one month before dialysis onset. These studies are more than 30 and 20 years old, respectively, and access to specialized care in Brazil has improved in recent years, but the population’s knowledge on CKD and nephrology as a whole has not advanced. A study carried out in Niterói, Rio de Janeiro, based on interviews with the population, showed that only 28% of people were aware of the word “nephrology”^7^.

People with CKD are known to have three times the risk of cardiovascular events compared to people without CKD. Therefore, patients with CKD are more likely to progress to death than to progress to end-stage renal disease^8^. CKD prevalence is estimated to be around 10% in the world population^1,9,10^. The main causes of CKD in the world are diabetes mellitus (DM) and arterial hypertension (AH), followed by glomerulopathies. Another factor that stands out, especially in low-income countries, are infectious diseases – the result of poor sanitation, poor quality water supply and high concentrations of disease-transmitting vectors. Furthermore, it is worth noting that there is a clear association between low levels of economic development and reduced availability of renal replacement therapy (RRT), such as hemodialysis, peritoneal dialysis and kidney transplantation, which increases the failures in the treatment of renal patients^1,11^.

Despite its increasing prevalence and relevance in world health, there is evidence of the lack of knowledge about kidney diseases, including patients with CKD, their caregivers and, above all, the elderly, who are generally more vulnerable to the main risk factors for this condition. This misunderstanding includes the most varied areas, in order to involve prevention, risk factors and available treatments, even in the current digital era, in which information technologies dominate the world scenario^7^. In a study carried out in Hong Kong including 516 participants, less than half of the sample knew that DM and AH were risk factors for CKD; 79.5% were unaware of the harmful effects of high-sodium consumption in the diet, and most of the sample believed that CKD symptoms were related to abdominal pain and urinary alterations^4^. In many studies, the highest rate of awareness about the disease is in those who already have an advanced disease, around stage 3, not to mention the fact that they do not receive adequate follow-up from a nephrologist^1,12^. The misunderstanding of this renal dysfunction can contribute to the demotivation and discouragement of patients to participate and adopt self-care measures to treat their comorbidities and, consequently, prevent the progression of the disease^8^.

Therefore, the objective of this study is to investigate the level of knowledge about CKD, its risk factors and means of prevention in the general population of a large urban area in Brazil.

## Methods

A cross-sectional study was carried out, through the application of a questionnaire to volunteers chosen at random in public places, in the city of Fortaleza, Ceará, Brazil, from November 2017 to January 2020. The interviews were carried out in public places, squares in the city of Fortaleza, and therefore the population sample is considered random. The population included in the study consisted of people over 18 years of age, of both sexes and with any level of education, excluding students and healthcare professionals, in addition to patients with kidney disease, because they already had presumably knowledge about CKD.

The sample size calculation was based on the population of Fortaleza, according to IBGE^13^ data (approximately 2,700,000 inhabitants), with a confidence level of 95% and a margin of error of 5%, resulting in an “n” sample of 385.

The subjects were invited to participate in an individual interview and to answer a semi-structured questionnaire (Appendix 1), after signing an informed consent form (ICF), which addressed topics such as: gender, age, origin, marital status, education, religion, occupation, as well as questions related to knowledge about CKD: “Do you have any type of disease?”, “Do you know what chronic kidney disease is?”, “Do you know what creatinine is?”, “Do you know what are the risk factors for chronic kidney disease?”, “Do you know the preventive measures for chronic kidney disease?”, “Would you like to get information about chronic kidney disease?” and “How do you get information about chronic kidney disease?”.

After completing the form, the interviewer explained each topic addressed to the interviewee and resolved any doubts related to the topic. Data were collected and then the information was stored in an Excel spreadsheet. The interviewers, all medical students between the third and fourth year of Medical School, were properly trained to apply the questionnaire at the beginning of the study, and each interview lasted an average of 10 to 15 minutes. The training consisted of meetings to clarify the questions in the questionnaire and how to apply them to the interviewees, so as not to influence the answers. Meetings were also held with the study team to assess what would be considered the correct answers for each item on the questionnaire, since these were “open-ended” questions.

Initially, after collecting sociodemographic data, respondents were asked about various factors related to CKD. The first question was: “Do you have any kind of disease?”. The responses were grouped into 5 categories (Hypertension, Diabetes, Cardiovascular and Other Diseases) as they are risk factors for CKD. Regarding the question: “Do you know what CKD is?”, in addition to the definitions in accordance with the literature, all answers that had terms related to CKD, such as, “Disease that needs hemodialysis” were considered correct, “Disease that raises creatinine”, “Incurable kidney disease”, “The kidney does not work properly” and “Difficulty filtering the blood”. When asked if they knew people who had kidney problems, the answers were selected, and only those who had first-degree relatives (parents, siblings and grandparents) were considered. Regarding the question: “Do you know what creatinine is?”, all the answers that had terms found in the literature concept were considered correct, such as, for example, “Type of blood test”, “Substance existing in the blood”, “Something that is evaluated in the blood test to evaluate the kidneys”, “It increases in the blood when the kidney is not working”. Regarding risk factors, the questionnaire already offered three options, which were not read by the interviewer, such as Hypertension, Diabetes and Obesity, and the category “Low Water Intake”, always reported by the participants, and which may indirectly affect kidney function. When categorizing the responses on disease prevention factors, two new variables were included: “Good Water Intake” and “Healthy Eating”, in addition to those existing in the research instrument (Practice of physical activity, Obesity Prevention and Diabetes Prevention). These two factors are directly related to good kidney health. Respondents were also asked what information they would like to have about the disease, and the answers were categorized into “Define the disease”, “Forms of prevention”, “Treatment”, “Symptoms” and “Risk factors”. The other questions were related to the use of technologies for self-care in health.

The study protocol was reviewed and approved by the Research Ethics Committee of the University of Fortaleza (Opinion No. 2,393,733/2018), and participation was by signing the consent form.

The data were analyzed by descriptive and inference statistics. Categorical variables were presented through absolute and relative frequency. For this, we used the statistical program Infostat version 2020e. In the inferential analysis, the variables on knowledge were established as dependent (knows how to define CKD, knows how to define creatinine, knows risk factors and knows ways of prevention) and variables were created from the existing ones for better statistical understanding, which were: 1) Gender, 2) Age group up to 60 years and >60 years, 3) Education (Illiterate, Elementary School, High School and Higher Education), 4) Previous diseases (SAH, Diabetes and CVD), 5) 1st and 2nd grade background with CKD. To analyze the relationship between the variables, Pearson’s chi-square test was applied, with a significance level of 5% (p < 0.05).

## Results

The present study had 735 volunteers, a quantity higher than the “n” sample initially calculated, with a mean age of 38.7 ± 15.9 years; 55.8% of which were female. Most respondents (78%) came from Fortaleza, 406 (55.4%) completed; 160 (21.8%) completed higher education; 150 (20.5%) had incomplete elementary education; and 17 (2.3%) were illiterate.

With regards to the concept of CKD, 282 respondents (38.3%) said they knew how to define the disease; however, after analysis, only 127 (17.2%) actually answered the question correctly.

As for the concept of creatinine, only 47 respondents (5.8%) answered the question correctly.

**Figure 1.**
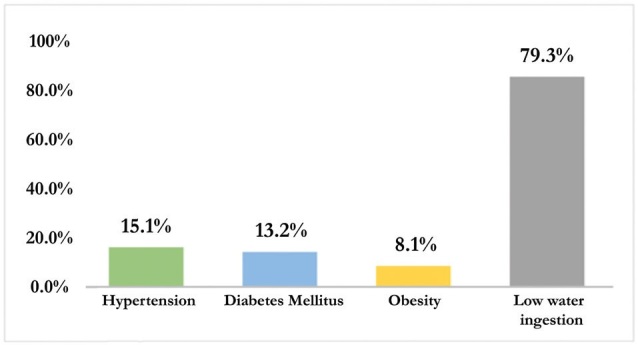
CKD risk factors properly recognized by the interviewees de risco para DRC corretamente reconhecidos pelos entrevistados. Fortaleza, Ceará, 2021.

Concerning risk factors for CKD, 320 respondents (43.5%) reported knowing such factors, but 19.7% got them wrong. Low water intake was the most cited risk factor by respondents (n = 204, 79.3%). However, the main risk factors and direct causes of CKD (DM and AH) were mentioned less frequently (13.2% and 15.1%, respectively), as shown in Figure 1.

**Figure 2.**
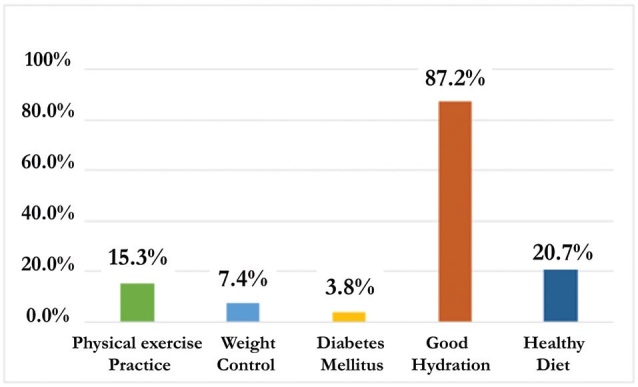
Ways of CKD prevention recognized by the interviewees. Fortaleza, Ceará, 2021.

Regarding the forms of prevention, most of the interviewees correctly recognized which factors can protect against CKD, so that, of the 400 people who reported knowing the forms of prevention, 367 (91.75%) actually got the factors right, such as shown in Figure 2.

It is important to highlight the low percentage of respondents who cited diabetes control, weight control, physical activity and healthy eating as a form of prevention, and none mentioned blood pressure control, as they are known to be directly related factors associated with the development of CKD.

Table 1 presents the assessment to knowledge about CKD, when relating it to some socioeconomic variables, such as gender, age group and education. There was a difference between genders in the understanding of the subject, with a higher percentage of men who got the definition, risk factors and forms of prevention for CKD right (n = 69; 17.9%, n = 129; 39,7%, n = 171; 52.6%, respectively). However, only in terms of knowledge about risk factors, although there is a slight percentual difference, the analyzes show that men know more about this topic, and this result is statistically significant.

**Table 1.** Evaluation of CKD knowledge according to gender, age and schooling among the general population (N = 735). Fortaleza, Ceará, Brazil, 2017–2020

Knowledge about CKD		Can define CKD		Can define creatinine		Know the risk factors		Know the ways of prevention	
Sociodemographic variables	N	Yes		Yes		Yes		Yes	
		n	%	n	%	n	%	n	%
Gender									
Female	410	69	16.8	28	6.8	128	31.2	196	47.8
Male	325	58	17.9	15	4.62	129	39.7	171	52.6
***p-value***		0.7172		0.204		***0.0167***		0.1932	
Age									
<60 years	658	105	16.0	36	5.5	235	35.7	337	51.29
>60 years	77	22	28.6	7	9.0	22	28.6	30	38.96
***p-value***		***0.0056***		0.2004		0.2136		***0.0419***	
Schooling									
Illiterate	17	0	0	0	0	2	11.76	2	11.76
Elementary School	150	14	9.33	6	4	35	23.33	52	34.67
High School	406	57	14.04	17	4.19	135	33,25	197	48.52
University Education	160	56	35	20	12.5	85	53.13	115	71.88
***p-value***		***<0.0001***		***0.0008***		***<0.0001***		***<0.0001***	

With regards to age, older respondents answered more correctly the questions about CKD definition (n = 22; 28.6%) and creatinine (n = 7; 9.0%), while younger ones showed greater accuracy concerning risk factors (n = 235; 35.7%;) and forms of disease prevention (n = 337; 91.8%;).

With regards to education, there was a statistically significant correlation in all questions (p < 0.05). Participants with higher education, 115 (71.9%), were more correct in all answers, showing a higher percentage of knowledge regarding prevention factors. Illiterates and people with elementary education had the lowest percentages in demonstrating knowledge about the definition of the disease (n = 0; 0 and 9%, and n = 14; 3%) and creatinine (n = 0; 0%; and n = 4; 4%), respectively.

Finally, Table 2 shows the assessment of knowledge about CKD, according to the existence of previous diseases and 1st and 2nd degree kinship history. The results show that people who had AH correctly answered most questions (definition of CKD, definition of creatinine and risk factors) when compared to those who did not have the disease. However, there was a slight difference between the percentages regarding knowledge about creatinine – the study showed that hypertensive people (n = 11, 11%) know more about this test, the difference being statistically significant (p = 0.0182). Non-hypertensive individuals showed to know better about the prevention factors, but there was no statistical difference in the result. Thus, this result shows that hypertensive people have greater knowledge about CKD and its risk factors. In the group of diabetics, it was shown that people who do not have the disease had more correct answers about the definition of CKD (n = 122; 17.78%), risk factors (n = 242; 5.3%) and forms of treatment and prevention (n = 348; 50.7%). However, in none of the results there was a statistically significant difference.

**Table 2.** CKD knowledge according to previous diseases and 1^st^/2^nd^ degree family history among the general population (N = 735). Fortaleza, Ceará, Brazil, 2017–2020

Knowledge about CKD		Can define CKD		Can define creatinine		Know the risk factors		Know the ways of prevention	
Doenças prévias	N	Yes		Yes		Yes		Yes	
		n	%	n	%	n	%	n	%
Hypertension									
Yes	100	22	22	11	11	36	36	46	46
No	635	105	16.5	32	5.0	221	34.8	321	50.5
***p-value***		0.1791		**0.0182**		0.8155		0.3975	
Diabetes									
Yes	49	5	10.2	3	6.1	15	30.6	19	38.8
No	686	122	17.8	40	5.8	242	35.3	348	50.7
p-value		0.1751		0.9330		0.5083		0.1059	
Cardiovascular disease									
Yes	32	2	6.2	3	9.4	5	15.6	14	43.8
No	703	125	17.8	40	5.7	252	35.9	353	50.2
***p-value***		**0.0915**		0.3850		**0.0190**		0.4745	
1^st^ and 2^nd^ degree family history of CKD									
Yes	124	35	28.2	15	12.1	62	50	75	60.5
No	611	92	15.0	28	4.6	195	31.9	292	47.8
***p-value***		**0.0004**		**0.0012**		**0.0001**		**0.0100**	

People who reported having high cholesterol, dyslipidemia and “heart problems” were included in the Cardiovascular Diseases (CVD) group. The results were similar to those of the diabetic group, since those who did not have the disease answered more correctly about the definition of CKD (n = 125; 17.9%), risk factors (n = 252; 35.9%) and forms of prevention (n = 352; 50.2%), with a statistically significant difference between knowledge about risk factors. With these results, the group that did not have CVD demonstrated to have better knowledge about the risk factors that can lead them to develop CKD.

Regarding background, those who had a 1st and 2nd degree relative (father, mother, siblings, grandparents) with kidney problems had more correct answers when compared to those who did not, with a statistically significant relationship in all parameters. This result shows that having a family member with kidney problems is a factor that can influence knowledge about CKD.

In addition, during the survey, participants were asked about the type of information they would like to receive about CKD, and only 4% said they were not interested. Regarding those interested, most indicated that they would like to know about prevention factors (n = 357; 8.6%), definition of the disease (n = 207; 28%) and treatment (n = 164; 22.31%).

## Discussion

The present study shows little knowledge about CKD in a sample of the general population of an important urban area in Brazil (Fortaleza, Ceará, fifth largest city in the country). The sample obtained is representative of the population, since it is greater than the “n” obtained by the sample calculation. According to IBGE data, the sample is similar to the general characteristics of the population of Fortaleza, which is composed mostly of people between 15 and 64 years of age (70%), with secondary education observed in approximately 39% of the population, and higher education around 11%^13^.

According to the data analysis, most of the interviewees proved to be lay people with regards to knowledge about CKD, since only 17% of the interviewees correctly defined the concept of the disease, similarly to what has already been described in the literature, in different regions in the world^14,15,16^, which reflects a population ignorance concerning this disease. Furthermore, the misinformation regarding the topic also significantly extends to its risk factors, which makes the situation even more alarming, considering that the main triggering reasons are often preventable and controllable, with changes to lifestyle, adoption of a healthy diet and regular physical exercise, which, if properly aware and controlled, has a high potential to reduce the onset and progression of CKD^17,18^.

In addition to this misunderstanding about the disease, attention is drawn to the public that had the most incorrect answers, which was the one with less education, in order to translate the great social influence on access to knowledge, which requires greater implementation of awareness campaigns on the subject in places and by strategic means, in an attempt to democratize access to this information, with a view to reducing the impacts of this misinformation. It is not by chance that CKD has a higher incidence among people with lower education and lower purchasing power, as evidenced in studies in Brazil and even in rich countries, such as England^19,20^.

Among individuals who had previous diseases, hypertensive individuals showed greater knowledge about CKD compared to non-hypertensive individuals, which may suggest that health education carried out among patients with AH has positive results. However, the low percentage difference between the two groups with regards to knowledge of risk factors and ways to prevent the disease shows that this awareness is still not enough, given that hypertension is one of the main risk factors for the development of CKD, and hypertensive patients should be intensively educated about its prevention.

Similar arguments can be made about diabetics and people with CVD. According to this population, the scarcity of information about CKD was remarkable, demonstrating the need for farther education for these individuals, whether made available in medical offices or in awareness campaigns about the disease.

Individuals with 1^st^ and 2^nd^ degree relatives with the disease, as mentioned, had higher knowledge about the disease, probably due to the fact that the greater the proximity and/or kinship with a person with the disease, the greater the curiosity about the comorbidity.

Finally, a relatively large percentage of the interviewees still lacks more information about CKD, being of fundamental importance a greater spread of information about the disease, through the promotion of awareness campaigns promoted by the Ministry of Health to the least favored socioeconomically, and by a greater search for knowledge through the internet, social networks, among other sources of information by those who have access. New technologies and the use of social networks have been applied to nephrology^21,22,23^ in an attempt to expand access to information about the various aspects of CKD for the general population, and it is expected that in the near future the population will have a better level of knowledge to minimize the damage caused by CKD and other chronic diseases.

This study contributes with evidence that there is still a low level of knowledge of the general population about CKD. Therefore, new healthcare education strategies are necessary, in order to obtain greater efficiency in disease prevention. The main limitations of the present study include: 1) low representativeness of the Brazilian population, since it was carried out in one city only, 2) prolonged time for carrying out the study, generating the possibility of bias, 3) absence of a validated instrument for the purpose of obtaining the level of health education of the Brazilian population.

## Supplementary Material

The following online material is available for this article:

Study: population knowledge about chronic kidney disease.
